# Walnut Oil Oleogels as Milk Fat Replacing System for Commercially Available Chocolate Butter

**DOI:** 10.3390/gels8100613

**Published:** 2022-09-26

**Authors:** Andreea Pușcaș, Anda Elena Tanislav, Andruţa Elena Mureșan, Anca Corina Fărcaș, Vlad Mureșan

**Affiliations:** 1 Department of Food Engineering, Faculty of Food Science and Technology, University of Agricultural Sciences and Veterinary Medicine Cluj-Napoca, 400372 Cluj-Napoca, Romania; 2 Department of Food Science, Faculty of Food Science and Technology, University of Agricultural Sciences and Veterinary Medicine Cluj-Napoca, 400372 Cluj-Napoca, Romania

**Keywords:** wax, monoglyceride, walnut oil oleogels, structural properties, chocolate butter

## Abstract

A breakfast spread named chocolate butter exists on the market. For economic and technological reasons, cream in the original recipe is replaced with vegetable oils such as palm oil or by partially hydrogenated sunflower oil. The study aims to reformulate chocolate flavor butter, using cold pressed walnut oil (WO) oleogels (OGs) structured with 10% waxes and monoglyceride (MG), as a milk fat replacing system. The rheological, textural and microscopic characteristics of the oleogels and the spreads were compared. Oil binding capacity (OBC) and colorimetry were also assessed. Fourier transform infrared studies were used to monitor the composition of the samples. Oleogels and oleogel based chocolate butter behaved like strong gels (G’ > G”). The use of candelilla wax (CW) led to the formation of a much firmer spread (S-CW), with a hardness of 3521 g and G’_LVR_ of 139,920 Pa, while the monoglyceride-based spread (S-MG) registered a hardness of 1136 g and G’_LVR_ 89,952 Pa. In the spreadability test, S-CW registered a hardness of 3376 g and hardness work of 113 mJ, comparable to the commercially available chocolate butter. The formulated spreads exhibited shear thinning effects, and increased viscosity with decreasing temperature. A large round peak at 3340 cm^−1^ was present in the spectra of the candelilla wax-based oleogel (OG-CW) and the reference spreads due to hydrogen bonding, but was absent in S-CW or S-MG. The FTIR spectra of the alternative spreads exhibited the same peaks as the WO and the oleogels, but with differences in the intensities. S-CW exhibited a dense crystal network, with spherulitic crystals of 0.66–1.73 µm, which were statistically similar to those of the reference made from cream (S-cream). S-MG exhibited the lowest stability upon centrifugation, with an OBC of 99.76%. Overall, both oleogel-based chocolate spreads can mimic the properties of the commercially existing chocolate butter references.

## 1. Introduction

On a global basis, consumers’ preferred breakfast spreads vary considerably from region to region. In the former soviet countries (Russia, Moldova, Ukraine, etc.), the product called chocolate butter is very popular among the breakfast spreads and confectionary products. Its composition consists of pasteurized cream, sugar, cocoa, buttermilk, flavoring agents and antioxidants, but for economic and technological reasons, cream in the original recipe is replaced or mixed with vegetable oils such as palm oil or partially hydrogenated sunflower oil, leading to the usage of other ingredients such as defatted recombined milk, emulsifiers and coloring agents. Palm oil has become a common milk fat substitute in many dairy products (as have analogues) [1,2], decreasing the production and selling cost, but raising concerns related to sustainability, and carbon emissions in tropical areas due to deforestation and expanding palm trees plantations [3]. The high amount of saturated fatty acids in the composition of chocolate butter or similar breakfast spreads (from 20 up to 70% of the total fats) raise concerns related to their production and consumption, since recent regulations limit the saturated fatty acids presence in food products to as low as possible [4]. This is because upon consumption, they lead to increased serum levels of low-density lipoprotein (LDL cholesterol), favoring the development of cardiovascular diseases or some types of cancer, which might in turn proliferate due to the energy furnished by palmitic acid to the tumor cells [5].

Reducing the content of saturated fats is possible by reformulating the products with unsaturated fats or carbohydrates, strategies which might lead to altered or undesired properties and effects in terms of nutrition, oxidative status, flavor, texture and consumer’s acceptance [6,7]. Moreover, the usage of carbohydrates as fat replacers do not decrease the chances of developing cardiovascular diseases. Thus vegetable oils rich in polyunsaturated or monounsaturated fats are highly desired as ingredients [8]. Recent studies demonstrate that dairy products with modified compositions resulting from mixing the milk fat with other fats lead to reduced risks of developing cardiovascular diseases in comparison to those containing only milk fat [8]. For this reason, it might be hypothesized that its total replacement would increase the chances of combatting the negative health related implications of saturated fats. Currently, the Codex Alimentarius standard for dairy spreads (CXS 253-2006) imposes a milk fat content of at least 10%, thus the full replacement of the milk fat would lead to the development of a confectionary and not a dairy spread [9].

Formulating food products low in saturated fats and rich in poly or monounsaturated fatty acids originating from vegetable oils or fish oil is currently possible due to the oleogelation mechanism, which structures the liquid oils into semi-solid structures with the usage of edible structuring agents and imparts to this novel system properties similar to solid fat [10]. Some structural arrangements occur during oleogel (OG) formation due to the presence of self-assembly molecules, crystalline structures, or polymers that entrap the liquid oil and limit its flowability [11]. Oil-in-water model emulsions containing oleogels instead of the oil phase have also been developed with comparable physical stability to emulsions of anhydrous milk fat [12]. Oleogel-based dairy products have also been developed and studied, stressing the need to elaborate alternatives for dairy fat. A recombined whipped cream with improved nutritional profile was developed from emulsionated fat model systems, containing anhydrous milk fat mixed with various amounts (15%, 30%, 45%) of rice bran wax (RBW) oleogels [13]. A 20–22% reduction of the content of saturated fat in a commercial cheese was achieved by replacing milk fat with soybean oil gelled with 0.5–1% waxes [14]. Cream was fully replaced in an ice cream formulation with sunflower oil oleogel formed by a 12% mixture of phytosterols and oryzanol, and resulted in a good quality product [15]. Moreover, recent studies have shown that oleogel consumption, in comparison to liquid oil, will not lead to increased triglyceride levels in the blood after consumption [16,17].

In the present study, cold pressed walnut oil was structured with waxes or monoglyceride in order to be included as a fat replacing system in a spread, macroscopically and texturally similar to the so called chocolate butter. The technical aspects of the product are described in GOST 6822-67, introduced in 1968 by the Ministry of the Meat and Dairy Industry of the USSR and its characteristics are a sweet taste and an aroma of chocolate and vanillin, with a dense, homogeneous, plastic consistency, and without visible drops of moisture on the cut. Despite the CODEX STAN 87-1981 which stresses that a chocolate product should contain cocoa butter or other cocoa solids [18], the product still carries the name of “chocolate butter from sweet cream” despite containing only 2.5% cocoa powder.

Linoleic acid (Omega-6) is predominant in the composition of walnut oil (50–60%), followed by oleic acid (Omega-9) and linolenic acid (Omega 3) (8–15%), their concentration depending on the processing methods and cultivars [19]. Recently, both crude and refined, but also hot or cold pressed, or solvent-extracted walnut oil have been structured with monoglycerides, and the resulting oleogels were found to have different characteristics, due to different minor components in the oil [20]. 

The aim of the current study was to develop a chocolate spread based on walnut oil oleogel, given the recommendations in regard to the decrease of consumption of saturated fatty acids [21] and the nutritious compounds it would impart to the product. In addition to its fatty acid profile, walnut oil was chosen as a dispersion medium because it would impart to the spread a ”nutty” flavor and numerous biologically active compounds such as tocopherols, squalene, phytosterols and polyphenols [19]. These molecules could act as antioxidants during the thermal processing implicated in the oleogel formation and inhibit early lipid oxidation. A monoglyceride oleogel was also developed to explore the potential of manufacturing chocolate butter, since monoglycerides are already a common ingredient of spreads, but also in order to compare its suitability with that of candelilla wax. Wax- or monoglyceride-based oleogelation is a feasible method due to the simple processing method, the edible character of the structuring agents and their efficiency even in low concentrations. Having thus established the physical properties of the formulated oleogels, which are proposed as the fat replacing system, the oleogel-based spreads were then compared to two commercially available spreads: one made solely with cream and one with a blend of cream and vegetable oils (mostly palm oil).

## 2. Results and Discussion

### 2.1. Oleogels Elaboration

Oleogels with 10% candelilla wax (CW) and 10% monoglycerides (MG) were elaborated from walnut oil (WO) to be included in the formulation of the novel chocolate butter. This is still an acceptable concentration of structuring agents, given the results of M. Öǧütcüa and E. Yılmaza [22], who explored virgin olive oil structured with 10% wax as novel spreads, and those of C. Palla et al. [23] who optimized the composition of high oleic sunflower oil and monoglycerides (6-10-14%) oleogels to be suitable as margarines. 

### 2.2. Textural Analysis

#### 2.2.1. TPA Test

The candelilla-based walnut oil oleogel (OG-CW) and the corresponding spread (S-CW) exhibited higher values of hardness (5010.80 g and 3521.00 g, respectively) than the monoglyceride-based samples. An increase in the hardness was registered for S-MG (1474 g) in comparison to OG-MG (761 g), while for the samples containing candelilla wax, the oleogel had a firmer structure than the spread. It seems that in the case of the monoglyceride-based samples, the addition of sugar enhanced the hardness of the structure. The structure of the wax-based sample is given by physical forces such as Van der Waals or hydrogen bonding, which are weakened by the sugar moieties; by contrast, in the monoglyceride-based samples, the structure formation is similar to interesterification occurring between different mixtures of fat [24]. The textural parameters and the oil binding capacity of the 10% wax- and monoglyceride-based oleogels and the novel corresponding spreads are presented in Table 1.

However, in the alternative spreads designed as chocolate butter replacers, the concentration of the structuring agent is as low as 7.5%. The high values obtained for the hardness (5010.80 gfor OG-CW and 761.00 g for OG-MG) indicated the formation of strong, stable internal networks, which were confirmed by the high OBC.

C. Palla et al. reported a hardness close to 300 g for the 14% Myverol-based oleogels, and the 10% sunflower wax oleogels studied by M. Öǧütcüa and E. Yılmaza. registered a similar hardness (306.01 g). Both might be suitable as margarine replacers, since the hardness of margarine is reported to be close to 160 g [23], but not as butter alternatives, since commercial butter has a registered hardness of 809.10 ± 59.02 g. 

Chocolate spreads based on mixtures of palm oil with oleogels, sugar (50%) and cocoa (10%) have also been proposed as healthier spread alternatives and, in the textural analysis, a 5% MG-based sample registered higher hardness than samples made with oleogels structured with the same concentration of beeswax or propolis wax [25]. Moreover, in the same study it, was revealed that during storage, the hardness of the wax-based spreads might increase, while that of the MG-based sample might decrease.

Because of the sample preparation, the TPA test was not applied on the commercially available spreads, the oleogel-based spreads being poured after manufacturing in to cone-shaped plastic cups (45 cm height × 55 cm diameter), just as their corresponding oleogels. The spreadability test was conducted to compare the textural performance of the oleogel spreads with the reference spreads.

The results in this test are in agreement with the findings of Athira Mohan et al. [26]: in the their study, CW oleogels displayed higher firmness and cohesiveness than monoacylglycerol oleogels, and it can be concluded that the influence of sugar and cocoa addition does not change the behavior of the oleogels from this point of view.

The adhesive force offers information about the behavior of a sample put in contact with a surface (such as the tongue or teeth of a consumer), and low values are desirable. The various oleogels registered a statistically similar adhesiveness. The spreads registered lower values of adhesiveness in comparison to the corresponding oleogels (11.85 mJ for S-CW and 1.75 mJ for S-MG). Adhesive force of OG-CW was also statistically similar to that of S-CW, thus the addition of sugar did not influence this parameter.

Gumminess is a textural parameter used to describe the masticability of semi-solid foods and the registered values were statistically different for the oleogels, while for the spreads a masticability of 365 g for S-CW and 83.5 g for S-MG were registered.

The ratio of the positive force areas under the first and second compressions is defined as cohesiveness. The test revealed that the oleogel samples were more cohesive in their internal structure than the corresponding spreads, which registered lower values of cohesiveness, namely 0.10 for S-CW and 0.05 for S-MG. The cohesiveness of OG-CW and OG-MG are statistically similar (0.22 for OG-CW and 0.20 for OG-MG).

#### 2.2.2. Oil Binding Capacity (OBC)

This is a measure of the oil migration, which is a frequent phenomenon in spreads and might be perceived as a quality defect by consumers. To assess the influence of the addition of sugar and cocoa to the oleogel, the OBC of the spreads and oleogels were compared. OG-CW displayed the higher OBC of 99.99%, followed by OG-MG, which registered an OBC of 99.94%. However, S-CW also registered a high value which was statistically similar to that of the oleogels (99.98%), while for S-MG, the centrifugal stability was lower (Table 1).

In the study of M. Öğütcüa and E. Yılmaza [27], in terms of OBC, the prepared oleogel of virgin olive oil with MG was better than that prepared with CW, and in the study of Kyeong-Ok Choi et al. [28], CW-based grape seed oil oleogels registered lower OBC than when structured with MG. In our study, the OBC of OG-MG was 99.94%, which is statistically lower than that of OG-CW. In the study of HaoSun et al. [22], lower OBC was determined for the cold pressed walnut oil oleogel structured with 10.70% monoglyceride (<90%). A lower OBC could indicate that the chocolate butter would soften once applied in a thin layer onto a surface.

#### 2.2.3. Spreadability

The spreadability of the novel products was assessed and evaluated based on the hardness (g) and hardness work (mJ), which resulted in a compression test. Samples are considered to exhibit good spreadability when both the values register lower values. S-MG registered a hardness of 4085 g and a hardness work of 113.4 mJ. S-CW exhibited the lowest value of hardness (3376 g) and hardness work (113 mJ). The values are similar to those registered for commercially available chocolate butter containing cream in its composition (S-cream), which registered a hardness of 3544 g and a hardness work of 155.8 mJ. The reference which contains 3% cream and 59% blend of vegetable fat (S-Veg) registered the highest values for the spreadability parameters, namely a hardness of 6653 g and hardness work of 310.6 mJ, and was the least spreadable between the analyzed samples. 

For an estimation of spreadability of plastic fats, other authors propose the evaluation of both firmness (hardness) and stickiness (adhesive force) obtained in the TPA test, since more force is required to pull back the probe as stickiness increases, indicating a possible difficulty in spreading [29]. The hardness of S-CW is of 3521 g and the stickiness of 833 g, while for the monoglyceride-based spread (S-MG), lower values of 1136 g and 284 g, respectively, were registered.

Given the results obtained in the current work, we consider the spreadability test to be a more reliable method for assessing the behavior of spreads when served on a slice of bread.

S. Bascuas et al. developed oleogel-based cocoa spreads, which registered higher values for the hardness (105–110 N) in the spreadability test. This imparted a less oily or wet macroscopic property to their novel product, which was similar in terms of texture to their control sample containing 100% coconut butter as fat phase [30].

### 2.3. Rheological Behavior

#### 2.3.1. Amplitude Sweep Analysis

The oleogels and novel spreads exhibited gel-like behavior (G’ > G”) which was maintained within an LVR domain, until a cross over point was reached G’ = G”. For OG-MG, the limit LVR was determined at a shear strain of 0.0359 when the G’ value reached 233,610 Pa, which is quite similar to OG-CW for which G’_LVR_ was 209,460 Pa at a shear strain of 0.0333 (data not shown).

The values of the elastic modulus (G’) of the spreads were lower in comparison to the corresponding oleogel, thus the presence of sugar and cocoa appeared to decrease the strength of the internal microstructure and to lead to the development of more shear sensitive systems. This could also have been due to the decrease in the fat crystals caused by the reduction of the oil phase quantity in the spread formulation in comparison to the oleogel.

The S-CW registered a G’_LVR_ of 139,920 Pa at a shear strain of 0.01, while S-MG also displayed a smaller LVR domain, with a G’_LVR_ of 89,952.5 Pa, determined within the same shear strain. The elastic modulus of the references, S-cream and S-Veg, were four-fold higher than those of the spreads designed in the current study, and the G’_LVR_ limit was reached within a shear strain of 0.01.

In the amplitude sweep analysis, the LVR limit of the walnut oil oleogels were determined as being 2.1 × 10^5^ Pa for OG-CW and 2.19 × 10^5^ Pa for OG-MG. In regard to the G’ and G” of the oleogels, similar values were reported by other authors in the case of 10% candelilla wax-based oleogels structured with other oils [31]. In the study of H. Sun et al. [20], similar high values of G’ (above 10^5^ Pa) were reached by different walnut oil oleogels structured with 10.7% monoglycerides, which were crystallized at room temperature and not under refrigeration conditions as in our study. However, spreads do not necessarily need strong gel-like behavior, because it might affect the spreadability of the samples. H. Sun et al. [20] revealed that the minor components and the type of the walnut oil (extracted, cold pressed, refined, etc.) influences the structural properties of monoglyceride-based oleogels. 

**Figure 1:**
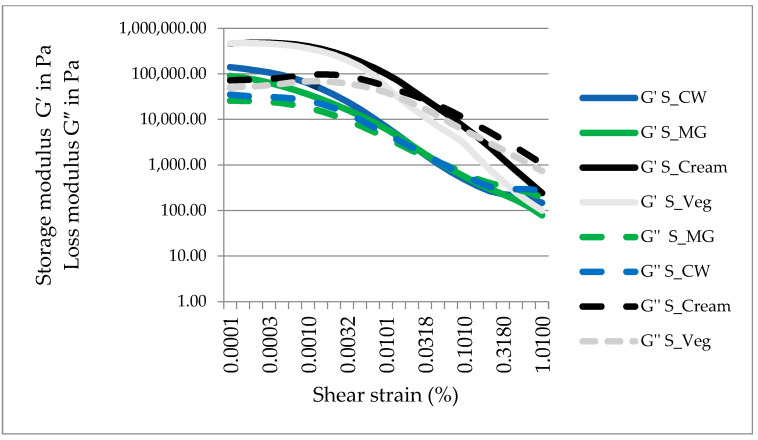
Amplitude sweep measurement of oleogel spreads and the references.

A crossover point where the G’ = G”, after which the structure of the sample is broken by the applied shear forces, was registered for each sample, as seen in Figure 1. As similar LVR regions were determined for the oleogel-based spreads, their cross over points were registered at 1695 Pa for S-CW and 1140 Pa for S-MG at shear rates between 0.03–0.04. The cross over point of S-cream was also determined at a shear rate of 0.03, where its G’ = G” = 27,015 Pa, but the reference spread which contained vegetable fats, S-Veg, registered the cross over point at lower shear rates (0.0216), where the G’ = G” = 30,210 Pa.

The sugar crystals strongly interact with vegetable oils, in the presence mostly of emulsifiers but also of monoglycerides, causing a better dispersion of the sugar particles in the oil and resulting in a viscosity decrease [32]. This is in agreement with our results and the decrease of elastic and plastic modulus registered for the spreads in comparison to their corresponding oleogels.

#### 2.3.2. Frequency Sweep Analysis

During the frequency sweep test, the storage modulus of the spreads dominated over the loss modulus. The G’ increased slightly over the frequency range, but with low dependence to the frequency, all the samples having a semi-solid behavior with good tolerance to deformation. C. Palla et al. [23] demonstrated that the monoglyceride-based oleogel preparation temperature (TP), the speed of agitation (SA) and the ambient cooling temperature (TC) influence the dependence of the structure on the frequency applied in the rheological test and their solid-like behavior.

**Figure 2:**
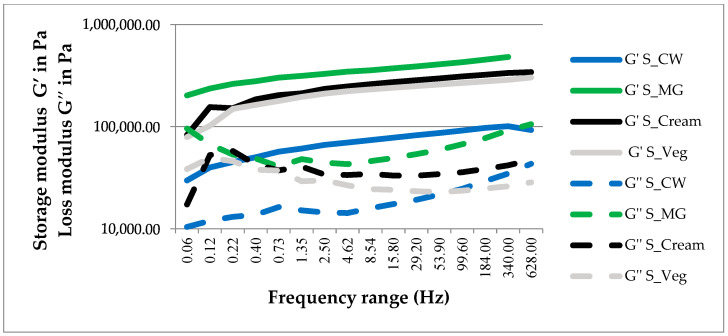
Frequency sweep measurement of oleogel spreads and the references.

The S-MG spread exhibited highest values of G’, followed by the commercially available spreads, as seen in Figure 2. In regard to the complex viscosity, the same trend was observed with highest values for S-MG. The complex viscosity of the samples decreased during the frequency sweeps, revealing shear thinning effects. The resistance to syneresis can be evaluated through the loss factor (G”/G’), the highest range being reached by the S-Veg with a maximum value of 0.501, followed by the oleogel spreads with a maximum loss factor of 0.468 for S-CW and 0.4825 for S-MG, and finally the S-cream (0.3755).

The frequency sweep test revealed that the novel spread would present the same good stability during long period storage as the commercially available samples. Also, the results indicate that the novel oleogel spreads could be produced on an industrial scale and would not be destabilized during processing, distribution or shelf-life.

Other authors who formulated chocolate spreads with sunflower or olive oil oleogels structured with HMPC and xanthan gum, registered lower values for the G’ and G” of their spreads (with values between 10^3^–10^4^ Pa) and with frequency-dependent properties, denoting their shear thickening character and lower stability [30].

Chocolate spreads with 50% palm oil and 50% oleogel structured with 5% monoglycerides present no frequency dependency in comparison to those elaborated from beeswax and propolis wax in the study of G. Fayaz et al. [25].

#### 2.3.3. Temperature Ramp

**Figure 3:**
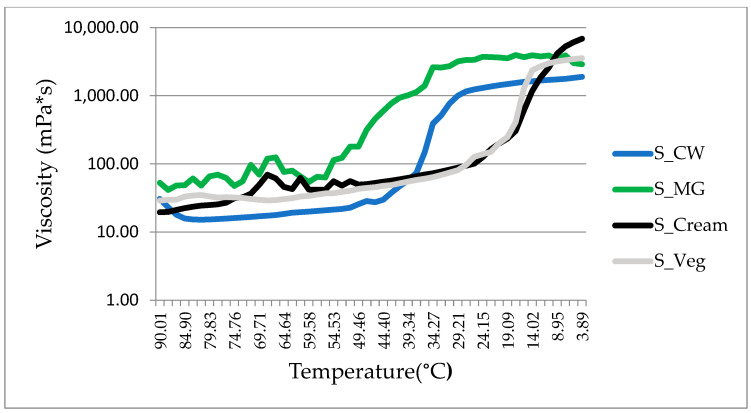
The determined viscosity of the oleogel spreads and the references at several temperatures within the range 90–4 °C.

The evolution of the viscosity during the cooling of oleogels and spreads was studied in order to determine the influence of sugar and cocoa on the crystallization behavior of oleogels. The viscosity of all samples increased in a non-linear way with decreasing temperature as seen in Figure 3. An abrupt phase transition was observed for both oleogels and spreads. The cloud points determined for the spreads are as follows: 52.9 °C for S-CW, while for the corresponding oleogel (OG-CW) the cloud point was registered at the lower temperature of 50.9 °C, thus the addition of cocoa and sugar promoted crystallization. For S-MG the cloud point was determined at and 57.6 °C, while for the corresponding oleogel (OG-MG) at 58.9 °C and a rather neutral effect is registered in the case of monoglyceride-based samples in terms of the crystallization temperature. The oleogel-containing spreads started to register positive changes in the viscosity at around 55 °C, their pour point being registered at 43.1 °C (S-MG) and 42.3 °C (S-CW).

The viscosity of S-CW at 4 °C is comparable to that of S-MG and S-Veg. The commercially available spreads display lower viscosities at 20 °C, which would impart them good spreadability, and even lower viscosities at 40°C, thus good flowability properties. The novel oleogel spreads registered high viscosities of 1425 mPa*s for S-CW and 3545.9 mPa*s for S-MG at 20 °C. For this reason, we tend to believe that the oleogel-based alternative spreads do not give a similar mouth-feel to that of milk fat-containing spread.

At 40 °C, S-MG registered high values of viscosity, namely 1015.98 mPa*s. This is due to the high melting temperatures of the monoglycerides, which maintain the structure of the three dimensional network that entraps the liquid oil. At 40 °C, S-CW presents viscosities similar to the reference spreads and good flowability.

In regard to the influence of temperature on the viscoelastic properties of the samples, other authors have revealed that G’ and G” of the 10% candelilla wax canola oil oleogels remained constant within the range of temperature lower than 40 °C, which might indicate that a more stable system might be developed from CW which does not require refrigeration in order to maintain its structure [33]. 

### 2.4. FTIR Analysis

**Figure 4:**
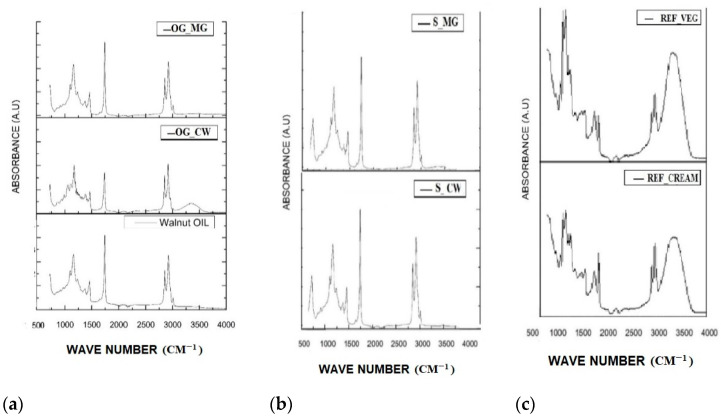
FTIR spectra of (**a**) oleogels and walnut oil, and (**b**) corresponding spreads; and (**c**) reference spreads.

FTIR was used to assess the impact of oleogelation or mixing oleogel with other ingredients (sugar and cocoa) on the microstructural properties of the samples. The acquired spectra are displayed in Figure 4. Hydrogen bonding is revealed in the OG-CW, because of the presence of a round large peak at 3340 cm^−1^. However, in the S-CW the peak is absent, thus the structure is not due to hydrogen bonding. Peaks at 3008 cm^−1^ are specific to the polyunsaturated fatty acids and are less visible in OG-CW. The peak at 2914 cm^−1^ corresponds to the methylene C-H asymmetrical stretching.

Absorption peaks due to C=O stretching appeared at 1752 cm^−1^ due to the ester bonds of the glyceride backbones which make up the oil. The absorbance in this region is decreased for the OG-CW and this might be due to interesterification. However, in the S-CW the peak has higher intensity, similarly to the walnut oil (WO). No differences in the intensity of this peak were observed for OG-MG and the corresponding spread (S-MG).

Weak peaks are present around 1650 cm^−1^, which can be assigned to the cis C=C stretching and are more prominent in the oil sample and the monoglyceride-based oleogel.

Peaks at 1459 and 1345 cm^−1^ are due to the stretching vibration of C-H in CH_2_ and CH_3_ groups which are the backbone of the fatty acids. The difference in the intensities occurred in this region between the walnut oil and the oleogels, the decrease of the absorbance revealing the differences occurring in the microstructure due to the oleogelation.

In the 1165 and 1000 cm^−1^ region in OG-CW, multiple peaks were registered due to the stretching vibration of C-O in C-O-H and in C-O-C. Peaks formed at 1145 cm^–1^ might also be due to the PO2 group vibration of the phospholipids present in the cold pressed walnut oil. The shoulder of the peak around 1000 cm^−1^ is not visible in the corresponding spread or in the walnut oil. The peaks at 719 cm^−1^ can be assigned to both CH_2_ rocking vibration groups of the fatty acids. 

In the commercially available references, a peak in the region 3000–4000 cm^−1^ was developed due to the O-H symmetric stretching of the emulsions. Cocoa powder was revealed by other authors to develop peaks between 3800–3000 cm^−1^, but also a sharp peak at 2800 cm^−1^ and multiple peaks in the 1800–1000 cm^−1^ region, depending on its fat, protein or moisture content [34]. These might be visible in the commercially available references. Peaks were also developed at 2918 cm^−1^ in the references, but with some differences in their intensity. These are due to the stretching of the C-O-C groups of the bond between glycerol and fatty acid ester carbon in triacylglycerol. Peaks at 1052 cm^−1^ display higher intensities for S-Veg in comparison to S-cream and might also be due to the fructose from the sugar in the composition. Pure sucrose determines the formation of peaks at 1049 cm^−1^ and 994 cm^−1^ [35].

In the 1095–1150 cm^−1^ region, peaks can be also due to the phytosterol esters of which walnut oil is reported to contain 973.7–2880.3 mg/kg of oil [36].

FTIR spectra of walnut oil oleogels developed from crude and refined oils and monoglyceride were previously reported by H. Sun et al. [20]. The structure of MG-based oleogels is mainly due to intermolecular hydrogen bonds between the OH groups which the monoglycerides contain and to C=O groups of fatty acids [20,37], but in the current study there was no peak in the 3000–3500 cm^−1^ wave range. In addition, sugar is reported to have free –OH groups, but this was not revealed in the spectra of novel oleogel spreads [25]. The forces which might govern the structure might be Van der Waal organization of alkyl groups, which are reported to exhibit peaks around 2900 cm^−1^, as registered for S-CW and S-MG [37].

### 2.5. Microscopy Analysis

**Figure 5:**
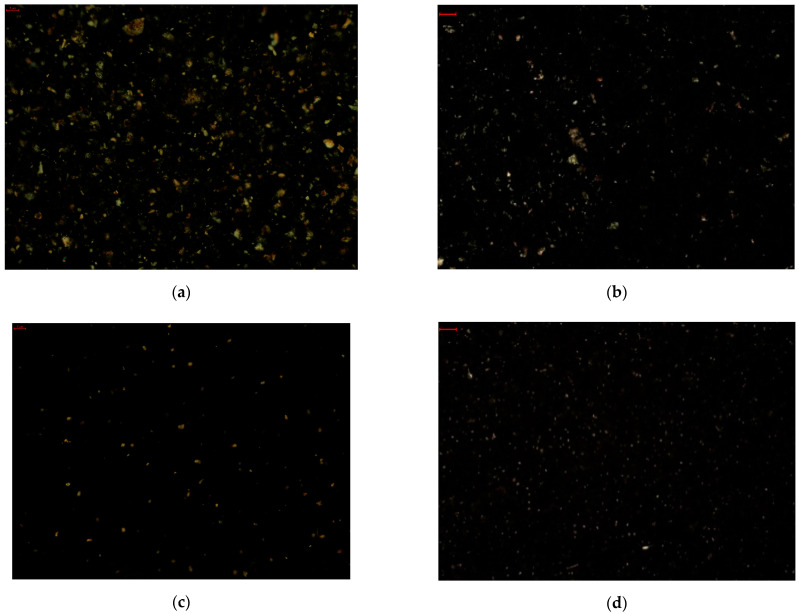
Microscopic appearance of spreads (**a**) S-CW, (**b**) S-MG, (**c**) S-Veg, and (**d**) S-cream in polarized light (4× magnification, scale bar dimension: 5 µm).

Micrographs of the spreads display the differences occurring in the microstructure of the samples due to their dominant ingredients. While the reference spread which contains milk fat exhibits spherulitic crystals, S-CW also exhibits spherulitic crystals which are typical for these oleogels. The monoglyceride-based spread exhibits needle-like crystals, just as has been observed for this class of oleogels by other authors. Thus the addition of sugar and cocoa does not alter the form of the crystals originating from the oleogel. As seen in Figure 5, the oleogel-based spreads exhibit a more dense structure in comparison to the reference spread containing cream. S-cream presents a looser network with round shaped holes, which might be the air included during the churning of the milk fat, stabilized by the fat crystals on the exterior.

The size of the crystals present in the microstructure of the oleogel-containing spreads and the references was determined. Each of the samples displayed various sized crystals, thus they were categorized as small crystals, medium length, and maximum length crystals. Similar classification was proposed by I. Szymańska et al. [38], in their study of candelilla oleogels as palm fat replacers.

The reference spread containing cream exhibited the smallest crystals in terms of maximum length (1.6 µm), being similar with the maximum length determined for S-CW. The reference spread containing vegetable oils presented the biggest crystals (3.64 µm), which were statistically similar only to the S-MG and the other reference. In terms of the medium crystals, statistically significant similarities were determined between S-CW and the reference spread containing vegetable oil, but also between the monoglyceride-based spread and the references. The smallest crystal lengths were determined in the novel wax-based spread (0.66 µm) and the reference containing cream (0.61 µm), for which no statistical difference was registered. The mean values of the dimensions determined for each spread are summarized in Table 2.

Other authors report that olive oil-monoglyceride organogels have needle-like crystals 5–15 μm in length (Kesselman and Shimoni, 2007) [25], and the ability of MG to form a gel from vegetable oils is associated with the formation of lamellar phases.

### 2.6. Colorimetry

Color parameters CIE LAB are studied in order to reveal some similarities between the oleogel-based chocolate spreads and the references in terms of color, which plays a significant role in the consumer’s product acceptance. Cocoa influences the color parameters, but since it is present in the same amounts in all the samples, the differences might occur due to the rest of the ingredients. The results are summarized in Table 3. 

The L* parameter represents the luminance (0–100): the candelilla wax-based walnut oil spread is similar in terms of this parameter to the reference spread which contains milk fat.

The same trend is observed for the whiteness index (Wi) of the chocolate butters. The monoglyceride-based spread S-MG presented increased luminosity and a Wi of 38.32. The Wi of S-CW was statistically similar to that of S-cream, being 22.09 and 22.45, respectively. The a* parameter represents the share of red color (>0): both the wax and monoglyceride-based spreads are statistically similar to the reference made from vegetable oils in terms of this parameter, S-CW having the highest (a = 3.46), followed by S-Veg (2.94), then S-MG. The b* parameter represents the share of yellow (>0) and again a significant similarity was found between the wax-based spread S-CW (b = 0.63) and the reference spread containing cream S-cream (b = 0.98).

The ∆*E* values were analysed in order to assess how the total color differences between the reference creams and each of the oleogel-containing spreads appeared to the human eye. When comparing the candelilla wax-based spread with the references, no statistically significant differences were obtained between the registered differences of this spread with the references (∆*E* of S-cream is 2.63 and ∆*E*S-Veg is 2.56). Also because ∆*E* < 5, no color differences might be observed by consumers. For the monoglyceride-based spreads, higher values of ∆*E* were determined, thus color differences might be perceived by consumers (∆*E*cream is 16.12 and ∆*E*veg is 13.36).

The color parameters of wax-based oleogels were compared to those of breakfast margarine and butter in the study of Yılmaz, E. and M. Öğütcü [39], and differed according to the oil used (hazelnut oil, olive oil). The study stated that coloring agents might be added in order to reach the desired appearance.

## 3. Conclusions

In the current work, besides obtaining a product with improved nutritional properties—a spread rich in polyunsaturated fatty acids, the influence of other food ingredients (sugar, cocoa) on the physical properties of the oleogels was revealed. As expected, mixing oleogels with other ingredients influenced firmness, OBC and caused some changes in the crystallization. Some differences in the microstructure were also revealed in the FTIR spectra.

Ripening and churning are processes to which milk fat is subjected in order to develop spreads with suitable melting and rheological properties by changing the polymorphic form, crystal size and the presence of water in the product. All these aspects might be further studied as processing technologies in order to improve the manufacturing of the novel oleogel-based spread.

Candelilla wax-based walnut oil oleogel is a good candidate for reformulating the conventional chocolate butter found on the market while diminishing the amount of saturated fats. S-CW registered a high firmness (3521 g) and low adhesiveness (11.85 mJ) and a spreadability comparable with the reference containing milk fat. It behaved like a strong gel (G’ > G”) and displayed good tolerance to deformation. The microstructure of S-CW in terms of crystal shape and size resembled that of the reference spread containing cream (S-Cream). The peaks exhibited in the FTIR spectra were in the same region as those exhibited by the walnut oil, but with changes in the intensities. Also, the candelilla wax-based oleogel spread exhibited good OBC (99.98%) and no statistically significant perceptible differences for color parameters in comparison to both reference spreads.

S-MG has also a great potential for developing alternative spreads, but exhibited some inconveniences such as the lower firmness, lower OBC (99.76%) and differences in the color parameters. In terms of microstructure, it presents the advantage of retaining a solid structure at higher temperatures and its microstructure is similar to that of the reference spread obtained from a blend of vegetable oils and cream.

## 4. Materials and Methods

### 4.1. Materials

Cold pressed walnut oil, icing sugar and cocoa powder were purchased from a local market. Candelilla wax (2039 L) was kindly donated by Khal Whax (Trittau, Germany). The waxes are natural according to the description provided by the producers and are stored in refrigerated conditions (0–4 °C). Glyceryl Monostearate Powder (>95.0% monoglyceride) was purchased from Alfa Aesar, Thermo Fisher Scientific, Kandel Germany. 

### 4.2. Manufacturing of Oleogels

Elaboration of the oleogels was done using a magnetic hot stirrer plate (IKA RCT, Staufen Germany) set to 450 rot/min. The wax and the glyceryl monostearate were dispersed in the oil and the resulting mixtures were heated above the melting temperature of each structuring agent (73 °C for the wax and 80 °C for the monoglycerides). When clear dispersions were achieved, the stirring and heating were ended, the systems were transferred to plastic vials and were subsequently cooled at refrigeration temperature (0–4 °C). The gelation was noted after 24 h by the tube flipping observation, and the gel state was established as soon as the samples did not flow under the influence of gravity.

### 4.3. Preparation of the Spreads

The novel spreads were developed by gently mixing icing sugar and 2.5% cocoa powder in with the oleogels immediately after the full dispersion of their components. The fat phase represents 77.5% while sugar 20%. The spread development was done in the RM 200 mortar, set at 1000 rot/min, in order to reduce the particle size dimension of sugar and waxes and to avoid unpleasant mouthfeel. The mixture was then poured in a mold and deposited at 4 °C to allow structure formation.

### 4.4. Textural Analysis

To assess the texture profile analysis (TPA) of oleogels and spreads, the CT3 Brookfield Texture Analyzer (5 mm target distance, 1 mm/s test and post-test speed, trigger load 1 g (Brookfield Engineering Labs, Middleboro, MA, USA) equipped with 10 kg load cell and the cylindrical probe (TA25/1000; D 25 mm) was used. 

The spreadability of the samples was assessed in the compression mode, at a target value of 15 mm, trigger load 10 g and the test speed set to 2 mm/s, with the use of male and female conical probes. The samples were placed in the inferior probe in a manner that avoids air inclusion and the surface was gently straightened with a knife prior to measurement [40]. Texture Pro CT V1.6 software was used for computing of textural parameters.

### 4.5. Rheological Analysis

Linear viscoelastic region (LVR) of the oleogels and spreads and the non-destructive deformation range were measured by small amplitude dynamic measurement using rheometer Anton Paar MCR302 (Anton Paar, Graz, Austria), equipped with a parallel plate geometry (PP25) and diameter of 25 mm, a Peltier system and Julabo water bath. Dynamic stress sweep measurements were taken at a frequency of 1 Hz at 4 °C with oscillatory stress varying from 0.01 to 100%. Frequency sweeps were also performed by applying the frequency range of 0.1–100 Hz in the LVR range.

The crystallization behavior was recorded with the method described by Wijarnprecha et al. [41] and the Anton Paar MCR302 rheometer was used. Sample viscosity was measured at 20 s^−1^ using a parallel plate geometry (PP25) with a diameter of 25 mm. The baseplates were initially heated at 90 °C, the samples were placed on it and then cooled at a rate of 1 °C/min from 90 to 4 °C (ramp linear). The distance between baseplate and geometry was set at 0.5 mm and the normal force at 0 N. Following the temperature ramp test, the software of the rheometer determined the pour point (at the inflection point of the viscosity curve) and the cloud point (the point where the slope of the viscosity starts increasing).

### 4.6. Fourier Transform Infrared Spectroscopy

The FTIR spectra of oleogels and spreads were acquired with Agilent Cary630FTIR (Agilent Technologies, Chelmsford, MA, USA) equipped with ATR Diamond sampling module. Samples were taken directly (consecutively, one by one) from the fridge and placed on the FTIR. Background sampling was undertaken and then spectra were scanned in the 4000–600 cm^−1^ wave number range with a resolution of 16 cm^−1^ and 64 scans. Spectra were analyzed with Origin PRO8 software (OriginLab, Northhampton, MA, USA).

### 4.7. Polarized Light Microscopy

The microstructures of the oleogels and spreads were observed with polarized light microscopy, done with a Kern OBE-1 microscope (KERN & SOHN GmbH, Balingen, Germany) under ×4 magnification. For each spread, 5 photos were recorded and the length of 25 crystals was measured after calibration, similarly to the method used by I. Szymańska et al. [38].

### 4.8. Oil Binding Capacity (OBC)

The oil binding capacity was achieved by subjecting the spreads to a centrifugal force according to the method described by Okuro et al. [42]. Approximately 5 g of each oleogels was introduced after melting into conical tubes with a screw cap, Falcon type (50 mL), crystallized at 4 °C and placed in the Hettich Universal 320R centrifuge (Andreas Hettich GmbH & Co. KG, Tuttlingen, Germany). These were centrifuged for 30 min at a maximum speed of 9000 rpm corresponding to a relative centrifugal force of 13.1 × 10^3^ g. The temperature during centrifugation was set at 4 °C, but because no oil release was observed, the experiment was conducted at 21 °C in order to observe the stability of the product under ambient temperature. After centrifugation, the released oil was drained by inverting the tubes for 30–35 min. The comparison between the mass of the sample before and after a centrifugation cycle allowed the determination of the oil loss as a percentage of the initial oleogel mass. The analysis of the oil binding capacity was performed in duplicate.

### 4.9. Colorimetry

The color measurement of oleogels and spreads was performed with the portable colorimeter NR0 (3NH, Shenzhen, China). The lightness L, a (−a greenness, +a redness) and b (−b blueness, +b yellowness) color parameters were measured. The instrument performs an automatic calibration (L = 0, a = 0 and b = 0). The values L, a, b were provided by the instrument software. The measurements were performed at five different points on the surface of the oleogel. The white index (WI Hunter) of the samples was calculated using the formula indicated by the study of Hasda et al. [43]. Total color difference (∆*E*) between the novel spreads and the references were calculated according to Mokrzycki, et al. [44] as previously applied by Szymańska et al. [38].

(1) $$∆E=\sqrt{{\left(Lo-L1\right)}^{2}+{\left(ao-a1\right)}^{2}+{\left(bo-b1\right)}^{2}}$$

*Lo*, *ao*, and *bo* are the reference color parameters and *L*1, *a*1, and *b*1 are the color parameters of the novel spreads.

### 4.10. Statistical Analysis

All analyses were carried out at least in duplicate and results are exhibited as the mean of the values obtained. An analysis of variance (one way ANOVA) was also applied and when the test returned a significant F-statistic, the Tukey test (α < 0.05) was used as a post hoc test for the analysis of differences using MINI TAB9.

## Figures and Tables

**Table 1:** Textural parameters and oil binding capacity of walnut oil oleogels and the novel oleogel spreads.

Textural Parameter/Sample	Hardness (g)	Adhesive Force (g)	Adhesiveness (mJ)	Gumminess (g)	Cohesiviness (g)	OBC
OG-CW	5010.80 ± 429.93 ^A^	891.00 ± 111.02 ^A^	28.60 ± 3.81 ^A^	1075.30 ± 77.43 ^A^	0.22 ± 0.02 ^A^	99.99 ± 0 ^A^
OG-MG	761.00 ± 87.68 ^D^	482.25 ± 53.01 ^B^	21.42 ± 1.98 ^A^	126.30 ± 16.56 ^C^	0.20 ± 0.02 ^A^	99.94 ± 0.1 ^B^
S-CW	3521.00 ± 341.52 ^B^	833.00 ± 94.75 ^A^	11.85 ± 1.03 ^B^	365.00 ± 40.30 ^B^	0.10 ± 0 ^B^	99.98 ± 0.1 ^AB^
S-MG	1474.00 ± 202.23 ^C^	397.00 ± 46.66 ^B^	7.5 ± 0.56 ^C^	83.5 ± 12.02 ^C^	0.05 ± 0 ^C^	99.76 ± 0.14 ^C^

For the same textural parameter, identical upper-case letters between each sample type indicate no significant difference (*p* > 0.05).

**Table 2:** Dimensions of the fat crystals visualized in the oleogel spreads and the references.

Spread Type	Small Crystal Size (µm)	Medium Length (µm)	Maximum Length (µm)
S-CW	0.66 ± 0.07 ^B^	1.11 ± 0.09 ^B^	1.73 ± 0.20 ^A^
S-MG	1.34 ± 0.19 ^A^	1.95 ± 0.36 ^A^	2.97 ± 0.35 ^AB^
S-cream	0.61 ± 0.07 ^B^	0.93 ± 0.08 ^B^	1.6 ± 0.21 ^AB^

The upper-case letters between each sample type within the same class indicate no significant difference (*p* > 0.05).

**Table 3:** Color parameter of the novel oleogel spreads and the commercially available samples.

Sample	S-CW-WO	S-MG-WO	S-Cream	S-Veg
L*	22.18 ± 1.83 ^C^	38.44 ± 0.88 ^A^	25.16 ± 0.64 ^B^	22.68 ± 1.05 ^C^
a*	3.46 ± 0.26 ^B^	2.20 ± 0.15 ^C^	4.02 ± 0.20 ^A^	2.94 ± 0.25 ^C^
b*	0.63 ± 0.04 ^B^	3.22 ± 0.21 ^A^	0.98 ± 0.02 ^B^	1.99 ± 0.15 ^C^
Wi	22.09 ± 1.89 ^C^	38.32 ± 0.81 ^A^	22.45 ± 1.17 ^C^	25.07 ± 0.75 ^B^

The upper-case letters between each sample type within the same parameter indicate no significant difference (*p* > 0.05).

## Data Availability

The data presented in this study are available on request from the corresponding author. The data are not publicly available due to privacy restrictions.
